# Evaluation of the marginal gap and the three-dimensional trueness of suprastructures generated from scanning two different substructure framework materials for full arch implant-supported restorations

**DOI:** 10.1186/s12903-025-07206-5

**Published:** 2025-11-28

**Authors:** Nourhan S. Emam, Nayrouz Adel Metwally, Mohamed Moataz Khamis

**Affiliations:** 1 Faculty of Dentistry, Zagazig University, Zagazig, AlSharqia Egypt; 2 Department of Prosthodontics, Faculty of Dentistry, Alexandria University, Alexandria, Egypt; 3 Department of Prosthodontics, Faculty of Dentistry, Alexandria University, Alexandria, Egypt; 4 Department of Prosthodontics, Faculty of Dentistry, Alexandria University, Alexandria, Egypt

**Keywords:** Marginal gap, RMS deviation, Trueness, Implant-supported prosthesis, Suprastructure, Substructure, Bar

## Abstract

**Background:**

This study aimed to assess and compare the influence of intraoral scanning of 2 different full-arch implant-supported frameworks on the marginal adaptation and the 3D trueness of the suprastructures generated from the scanned frameworks.

**Methods:**

Two milled maxillary implant-supported frameworks were used in this study. The frameworks were manufactured from 2 different materials, Group I: titanium and Group II: Poly-Ether-Ether-Ketone (PEEK). A desktop scanner (Medit MD-1D0410, Medit Corp) was used to scan each framework once creating a reference scan, then the frameworks are tightened inside the patient mouth and an intraoral scanner (Medit i700; Medit Corp) was used to scan each framework 10 more times. From each intraoral scan, suprastructures were designed and 3D printed. The horizontal marginal gap between the finish line of the framework and the suprastructure for each group by using a stereomicroscope (SZ1145TR; Olympus). Every suprastructure in each group was assembled on the framework, tightened to the model, and scanned (*n* = 10) by using a desktop scanner. The suprastructures were scanned and compared with the reference file to evaluate 3D trueness through color-difference mapping by using a software program (The Medit link compare tool, Medit Design v3.0.6, Build 286; Medit Corp).

**Results:**

Regarding the marginal gap, the titanium group had a significantly lower average marginal gap (70.99 ± 6.53) µm than the PEEK group (101.87 ± 21.51) (*P* = 0.0001). Regarding the 3D trueness of the 3D printed suprastructures, the titanium framework showed significantly less RMS value deviation from the reference scan (124.30 ± 23.39) µm than the PEEK framework (212.60 ± 54.76) µm (*P* < 0.001).

**Conclusions:**

Intraoral scans of titanium frameworks produced suprastructures with superior marginal adaptation and 3D trueness compared to PEEK frameworks. However, the average marginal gap in both frameworks was considered clinically acceptable.

**Trial registration:**

This trial, number NCT06423482, is registered at Clinical.gov. Date of trial registration 21/05/2024”.

**Supplementary Information:**

The online version contains supplementary material available at 10.1186/s12903-025-07206-5.

## Background

Many prosthetic options, designs, and materials are available for full-arch fixed prostheses. A CAD-CAM milled suprastructure that is passively cemented to bar infrastructure is a popular rehabilitation option for implant-supported full-arch screw-retained frameworks [1]. 

Supra and substructure designs are commonly used in full-arch implant-supported rehabilitation [2]. Milled titanium frameworks were considered as the widespread option with excellent biomechanical features [3–5]. Polymer-based frameworks, comprising high-performance polyetheretherketone (PEEK), have become popular because of their elastic bone-like elastic modulus, light weight, and suitable biomechanical properties [6–8]. 

Previous studies have scanned frameworks to obtain perfectly fitting suprastructures [9, 10]. A lack of accurate fit may result from material dimensional changes after milling, design-related errors that include unfavorable undercuts, or inadequate milling parameters [11–13]. This is routinely done in laboratories not only as a part of the fully digital workflow but also to assure the machining trueness when compared to the original design STL file to detect prosthesis misfits [8, 12]. 

There are 3 types of marginal misfits: vertical, horizontal, and absolute. The term horizontal marginal discrepancy refers to the horizontal marginal gap assessed perpendicular to the framework withdrawal path [14, 15]. Clinically acceptable marginal gap discrepancies range from 50 to 100 μm for CAD-CAM restorations [16–18]. Assessment of marginal discrepancies in the prosthesis is crucial as good fit prevents microleakage and avoids the prosthesis failure [17]. 

Accuracy is an essential factor in assessing the usability of printed objects [19–21]. The International Organization for Standardization (ISO) 5725 standard states that trueness and precision define ISO accuracy [22]. Precision refers to the extent of concordance among independently measured values in a controlled setting, indicating the repeatability of measurements in asingle group, whereas trueness denotes the alignment between a test result and a recognized reference value [22, 23]. 

The 3D trueness evaluation of a prosthesis has been widely evaluated by using either metrology-grade or non-metrology-grade 3D analysis software programs [24]. Some materials have proven more scannable, true, and precise than others [25, 26]. 3D overall trueness is calculated by using the root mean square (RMS) method, which has been extensively used and interpreted by color-coded maps. The RMS method allows the deviations between the aligned virtual models to be 3-dimensionally quantified [25]. It is generally affected by parameters like printing errors, cement gap, internal fit, and marginal gap. Batson et al. [27] have reported the clinical outcomes of different crown systems for crown assessment with CAD/CAM technology, including the crown contour, marginal adaptation, shade, and occlusion, which states the influence of the marginal adaptation of the prosthesis and the overall trueness and longevity on its clinical outcome.

Occlusion was one of the most critical evaluation criteria [27]. Enhancements in the physical qualities and the accuracy of the internal and marginal fit contribute to prosthesis clinical success and overall accuracy [28–30]. 

There have been attempts to introduce 3D-printed definitive prostheses clinically. The physical properties of these materials have been improved to attain the strength needed for definitive prostheses determined by the International Organisation for Standardisation (ISO) standard 10 477 [31–33]. 

The purpose of the current study was to assess the influence of scanning 2 different framework materials on the marginal adaptation and the occlusal trueness of the suprastructures produced from those scans. The null hypothesis was that no significant differences between the groups would be found in marginal adaptation or occlusal trueness.

## Methods

### Patient and public involvement

The patient participated in the clinical phase of the study but was not involved in its design, outcome selection, data analysis, or interpretation. The purpose of the study was clearly explained, and the patient provided informed consent for participation and the use of clinical data in publication.

This clinical study was conducted following the CONSORT guidelines. The CONSORT checklist is provided as an additional file.

The primary outcome was the marginal gap (µm) of suprastructures generated from scanning titanium and PEEK frameworks. The secondary outcome was the 3D trueness, assessed by RMS deviation.

### Study design and patient selection

A maxillary edentulous patient with four integrated implants was selected for the study. The patient’s opposing mandibular arch was rehabilitated with porcelain fused to metal (PFM) full arch framework supported by 4 implants. The study was performed at the faculty of dentistry, Alexandria University.

### Framework fabrication

Multiunit abutments (MUA, Vitronex Elite) were selected correctly, tightened, and torqued to the implants for the maxillary arch. Scan bodies (MUA Scan body, Vitronex Elite) were tightened to the abutments following the manufacturer’s guidelines. A digital impression of the maxillary arch was obtained by using an intraoral scanner (Medit i700; Medit Corp).

A physical model of the virtual impression was 3D printed. A verification jig was designed and printed to verify the digital impression. The verification jig was designed, matched, and superimposed with the scanned jaw relation. Jaw relation was registered considering the opposing rehabilitated arch.

A fully anatomic try-in was printed and tried intraorally to verify esthetics and occlusion. The case design file was then split into a suprastructure and a bar substructure using Blender for Dental v3.6 (Blender Foundation, B4D iBar™ module) software program.

**Fig. 1:**
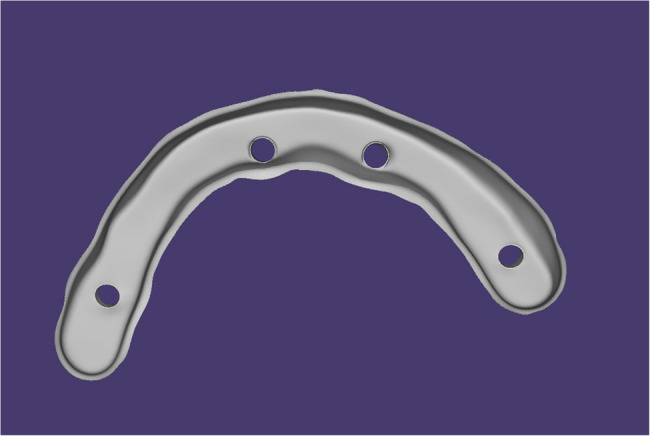
Bar design with buccal and lingual finish lines on (Blender for Dental v3.6; Blender Foundation, B4D iBar™ module) software program

A full-arch screw-retained framework with a maximum of one cantilever unit was designed for the patient [34, 35]. The inter-arch space and the arch width allowed for a bar height of 5.1–6.6 mm occluso-cervically, and a width of 5–6 mm buccolingually [35–37]. Buccal and lingual finish lines were created with a width of 1 mm, as shown in Fig. 1. A resin bar was 3D printed and tried intraorally before proceeding with the construction of the final bar.

Two groups in all were produced based on the type of framework material. PEEK (breCAM. BioHPP disk, Bredent GmbH & Co. KG) and titanium (dentatec, GmbH) [34]. The STL file of the virtual framework was exported for milling titanium and PEEK bars using a milling machine (Roland DWX-52D Plus, Roland DGA Corporation).

**Fig. 2:**
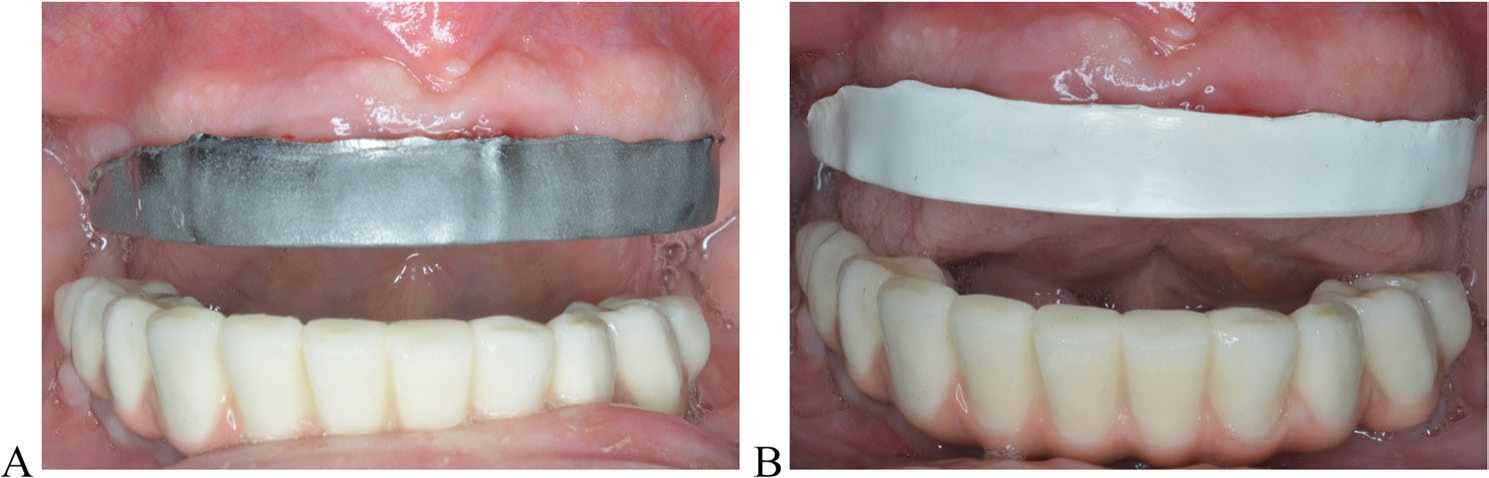
Bars try-in, **A** milled airborne particle abraded titanium and **B** milled airborne particle abraded PEEK

Airborne-particle abrasion was used to roughen both bars using 50 mm Al_2_O_3_ (Aluminum oxide, Eisenbacher Dentalwaren; ED GmbH). Abrasion was completed at 0.2 MPa pressure from a 10 mm distance for 1 min [34, 35, 38, 39], presented in Figs. 2A and B.

### Suprastructure fabrication

Each framework was scanned on a 3D-printed model by using the desktop scanner (Medit MD-1D0410, Medit Corp) to create an independent reference file STL_R_ to be used as a reference for each group. The reference suprastructures were created by drawing the margin on the desktop scanned bar finish line using a design software program (exocad DentalCAD; exocad GmbH).

Each bar was scanned 10 times intraorally to create the test specimens using an IOS (Medit i700; Medit Corp), from which 10 suprastructures were 3D printed. The margin was drawn on the finish line on each STL file, and a suprastructure was created. To standardize the 3D position of all specimens, each specimen was aligned with the original bar design from the software program (Blender for Dental v3.6; Blender Foundation, B4D iBar™ module), the automatic alignment feature was utilized, and then the quality of the superimposition was verified by the color map. To standardize the anatomical form of all suprastructures, the original wax-up was aligned with the saved jaw scan OBJ file for each suprastructure, so that each suprastructure was calculated with absolute standardization between all specimens in both groups. The margin was drawn for each scan with unified parameters for both groups of 0.08 cement gap [40]. 

The STL design of the virtual suprastructure was exported for production by using a hybrid nanoceramic-filled resin for 3D printing a wide range of permanent and temporary dental restorations (Flexera Smile Ultra+) by using the 3D printer (EnvisionTEC 3D printer, Envision One cDLM, Perfactory^®^). Creating a total of 22 specimens, 11 for each group, 10 suprastructures from each bar, and a reference for each group for comparison. All specimens were printed and cured following the manufacturer’s guidelines and recommended printing parameters [41]. Post-processing-curing was done in the (Otoflash G171) with 1000 flashes for 5 min, without temperature control. A letter representing the group (T/P) and a number were engraved in each specimen.

### Marginal gap assessment

**Fig. 3:**
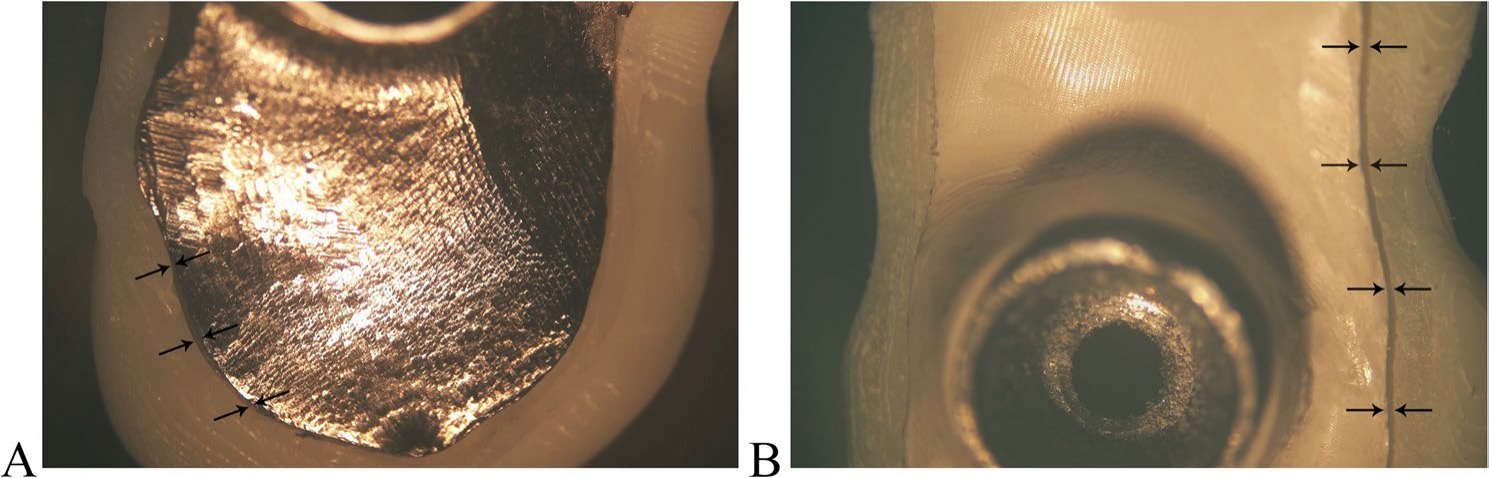
Measurement of the horizontal marginal gap in microns. **A** titanium bar, **B** PEEK bar

Each suprastructure was assembled on the bar and clamped using a customized clamp without cementation to measure the horizontal marginal gap in µm using a stereomicroscope (SZ1145TR; Olympus) at 25X magnification [42]. The stereomicroscope was equipped with a digital camera (HD Camera: XCAM1080PHB, Sony) and analyzing software (Fiji Imagej, version 2.14.0, NIH), an open-source software program for processing and analyzing scientific images [43]. presented in Fig. 3.

Marks were made at an equidistance on a standardized basis at 4 buccal and 4 lingual marks of each crown, for the whole suprastructure and on the entire sample size. Measurements at each point were repeated three times. Then, the average of all points was recorded and statistically analyzed. This consistent approach ensured reproducibility across all samples [40, 42, 44]. 

### 3D trueness measurement

Each suprastructure was assembled on either the titanium or the PEEK bar to be scanned with the desktop scanner 10 times in each group; _al_l the specimens were superimposed with the reference file of the desktop-scanned bar from which the reference suprastructure was created.

**Fig. 4:**
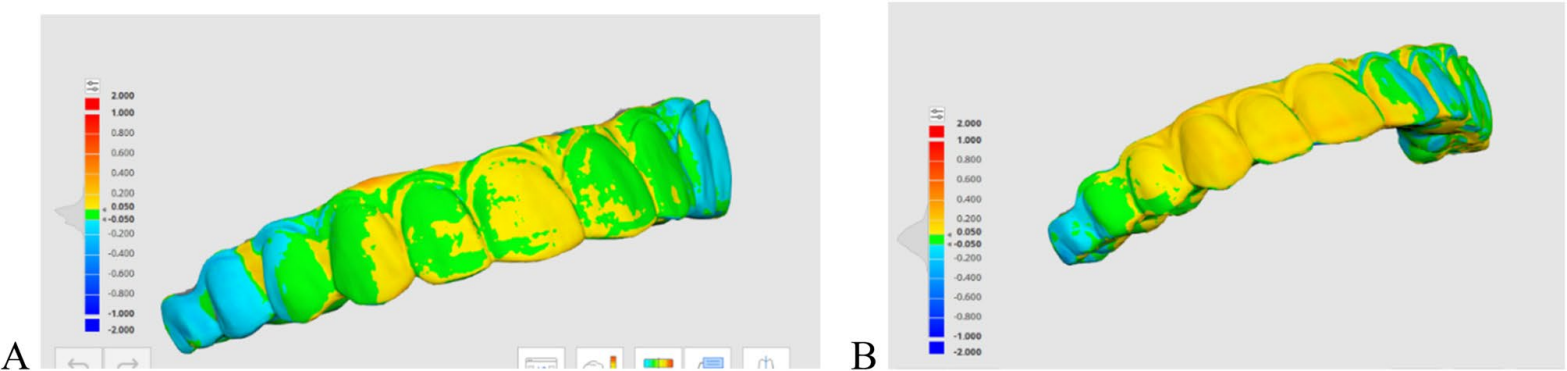
3D Trueness of the 3D printed suprastructures generated from **A** titanium bar, **B** PEEK bar, evaluated by color map to calculate the overall RMS deviations

The desktop scanned suprastructure file was utilized as the reference specimen to assess trueness by measuring the mismatch with the corresponding 10 suprastructures generated from intra-oral scanning of the bars in each group. Each reference file was aligned with the intra oral scan test file with the automatic alignment algorithm. The Medit link compare tool (Medit Design v3.0.6 Build 286; Medit Corp) was used for superimposition and measurements. First, the reference suprastructure file was moved to the reference data, and the test suprastructure file was transferred to the target data using the software program’s automated alignment function. The software program’s deviation display mode was used to produce color-difference maps to quantitatively evaluate 3D deviations between the reference STL_R_ and the target data STL_I_. The maximum and minimum deviation values were set at + 50 and − 50 μm (green) [25]. The software program automatically determined the overall RMS values (trueness) (on the lower left panel, among other data). The color-difference maps were used to calculate the overall RMS deviations and were converted from mm to microns. When a low RMS was present, a high degree of 3D matching of the superimposed data was obtained, indicating high trueness and vice versa [45] as shown in Fig. 4.

**Fig. 5:**
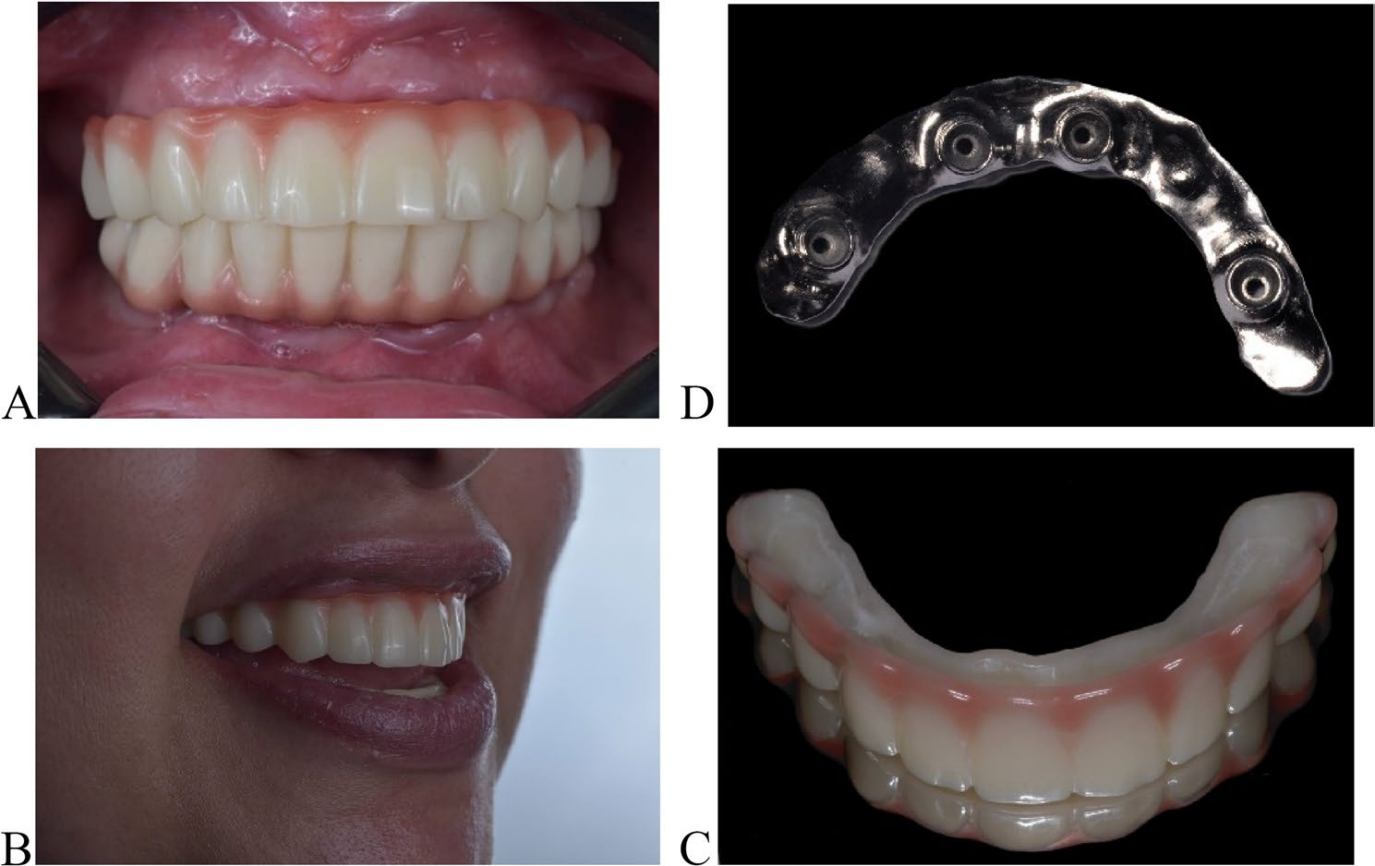
Final restoration. **A** Intraoral view, **B**, **C** Extraoral views. And **D** displays the polished intaglio surface of the bar

The patient was eventually restored with the titanium bar and 3D-printed suprastructures. The gingival part of the suprastructure was coated with a pink-colored composite resin (Visiolign; Bredent GmbH) to display natural esthetics. It was cemented with a self-curing composite cement multilink hybrid abutment (Ivoclar Vivadent) as shown in Fig. 5A, B, C, and Fig. 5D displays the polished intaglio surface of the bar. This trial is registered in Clinical.gov, NCT06423482.

### Statistical analysis

The sample size was estimated assuming 80% study power and 5% alpha error, using estimates reported by Kordi et al. [42], reported mean (SD) marginal gap = 13.3 ± 1.5 μm in preparable abutments and 21.3 ± 7.4 μm in titanium base abutments. Based on a comparison of means using a 2 tailed test, the minimum sample size was calculated to be 9 per group, increased to 10 to compensate for laboratory processing errors, with an effect size of (2.382). The total required sample size = number of groups ×number per group = 2 × 10 = 20. The sample size was calculated using G*Power (Version 3.1.9.7).

Normality was tested using descriptive statistics, plots (histograms and boxplots), and the Shapiro-Wilk normality test. All data showed normal distribution, so parametric tests were used. Descriptive statistics were calculated as means, standard deviation (SD), median, interquartile range (IQR), and 95% confidence interval (CI). Comparisons between the two study groups were performed using an independent samples t-test, calculating mean differences and 95% confidence intervals. The significance level was set at *P* < 0.05. Data were analyzed using IBM SPSS for Windows (Version 26.0). Blinding and randomization were not feasible due to the differences in the interventions; the operator inevitably knows which bar is being tested.

## Results

**Fig. 6:**
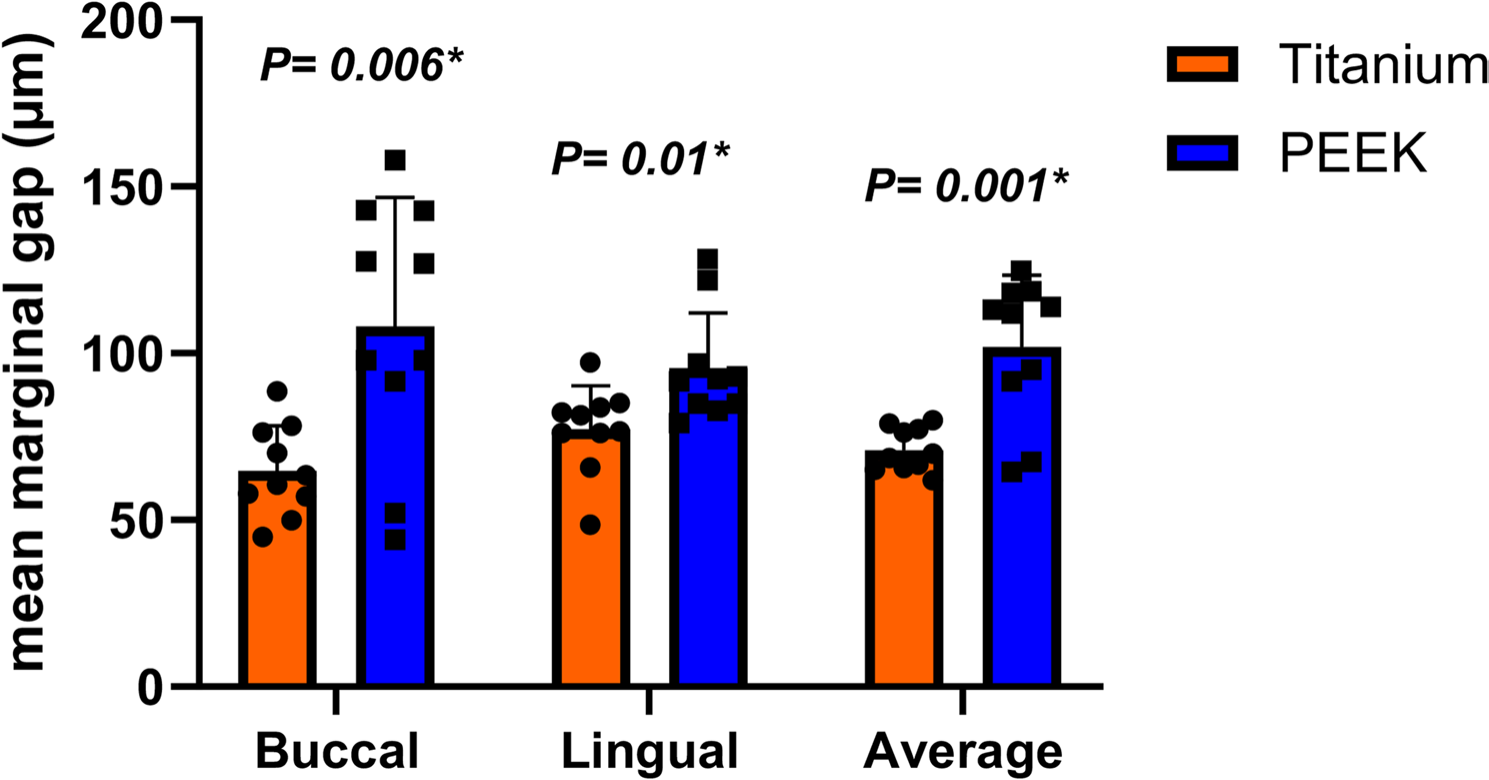
Marginal gap (µm) in the bars of the two study groups (titanium and PEEK)

Regarding the marginal gap results, the independent samples t-test was presented in Table 1. Mean± (SD)µm revealed that the titanium group had a marginal gap of (64.72 ± 13.54)µm on the buccal finish line and a marginal gap of (77.27 ± 12.92)µm on the lingual finish line, with an average of (70.99 ± 6.53)µm for both. As for the PEEK group, the buccal marginal gap was (108.15 ± 38.52)µm, while the lingual marginal gap was (95.58 ± 16.52)µm, with an average of (101.87 ± 21.51)µm for both. Suprastructures created from scanning the titanium bar have shown significantly less marginal gap than suprastructures created from scanning the PEEK bar, with T = 4.34 and *P* = 0.001 shown in Fig. 6.

**Table 1:** Marginal gap (µm) in the two study groups (titanium and PEEK) Bars

		Titanium	PEEK	Mean difference (95% CI)	*P* value
Buccal	Mean (SD)	64.72 (13.54)	108.15 (38.52)	−43.44 (−71.80, −15.08)	T = 3.36 P = 0.006*
	95% CI	55.03, 74.41	80.60, 135.71		
Lingual	Mean (SD)	77.27 (12.92)	95.58 (16.52)	−18.31 (−32.24, −4.37)	T = 2.76 P = 0.01*
	95% CI	68.03, 86.51	83.76, 107.40		
Average	Mean (SD)	70.99 (6.53)	101.87 (21.51)	−30.87 (−46.58, −15.16)	T = 4.34 P = 0.001*
	95% CI	66.32, 75.66	86.48, 117.25		

Independent samples t-test was used

*SD *Standard deviation, *CI* Confidence interval

*Statistically significant at *p*-value < 0.05

**Fig. 7:**
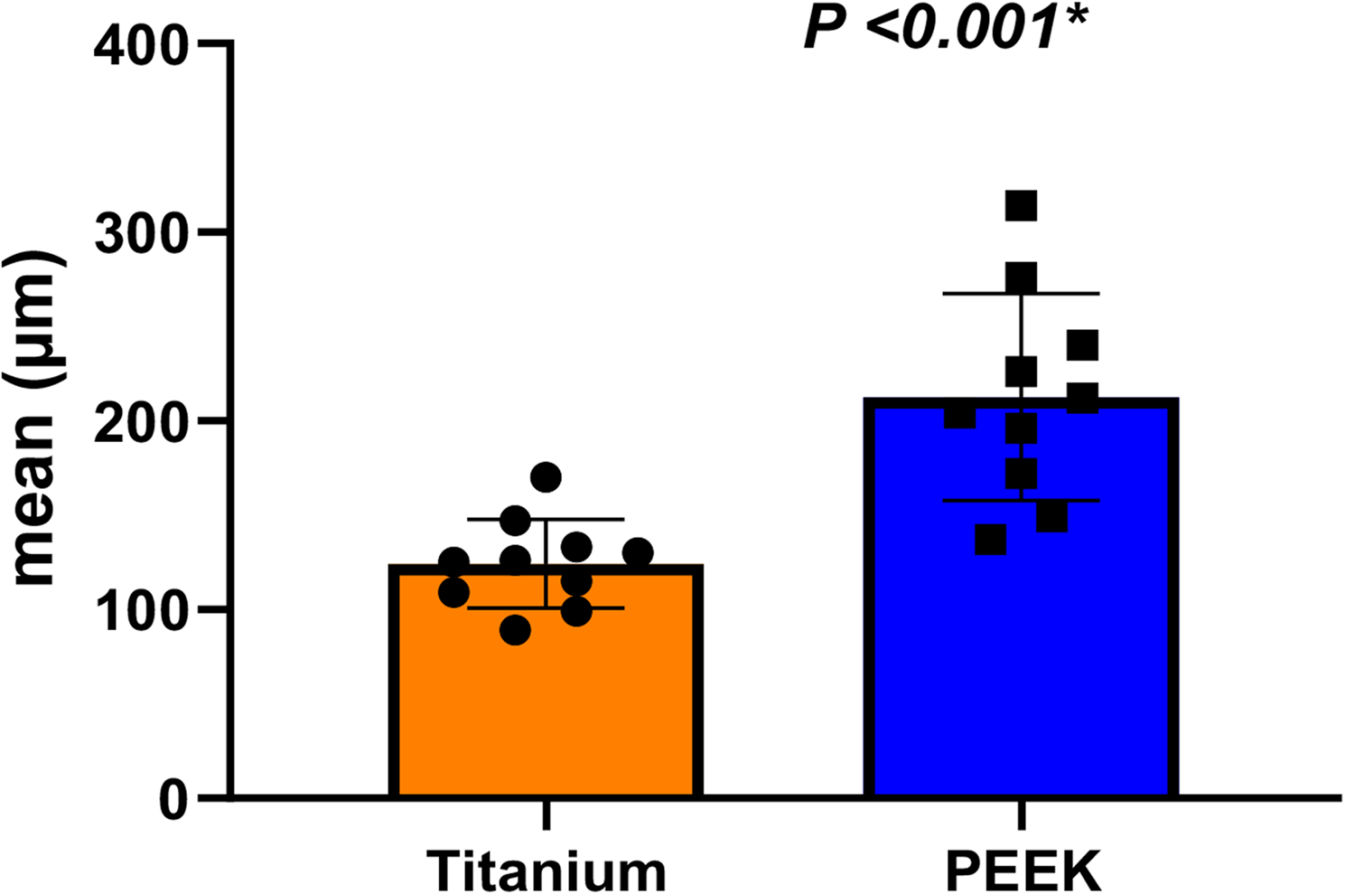
Measurement of 3D Trueness of the 3D printed suprastructures. Deviation in RMS (µm) in the two study groups (titanium and PEEK) bars

Measurements of 3D Trueness of the 3D printed suprastructures were represented by the RMS of the overall deviation as presented in Table 2. Suprastructures created from the titanium bar deviated from the reference specimen with (124.30 ± 23.39)µm. In contrast, the 3D trueness of suprastructures created from the PEEK bar was (212.60 ± 54.76)µm, which is significantly greater than the Titanium bar suprastructures’ deviations T = 4.69 and (*P* < 0.001), as shown in Fig. 7.

**Table 2:** 3D trueness of the 3D printed suprastructures (µm) in the two study groups (titanium and PEEK) bars

		Titanium	PEEK	Mean difference (95% CI)	*P* value
Deviation	Mean (SD)	124.30 (23.39)	212.60 (54.76)	−88.30 (−127.86, −48.74)	T = 4.69 P < 0.001*
	95% CI	0.11, 0.14	0.17, 0.25		

Independent samples t-test was used

*SD* Standard deviation, *CI* Confidence interval

*Statistically significant at *p*-value < 0.05

## Discussion

This research investigated the influence of scanning different bar materials on the marginal adaptation and 3D trueness of the printed suprastructure. The scanned substructure material type impacted the marginal adaptation and the 3D overall trueness of the printed suprastructure. Therefore, the null hypothesis for the marginal gap and the 3D trueness of the 2 different framework materials’ suprastructures was rejected.

This study aimed to assess a clinically relevant digital procedure that has been previously reported in the literature [39]. In the current study, during the clinical phase, intraoral scanning and bar data acquisition were performed to evaluate the influence of clinical scenarios and variables on the measured outcomes. During the laboratory phase, suprastructures were manufactured and analyzed under regulated laboratory settings.

Supra and substructure designs created by (Blender for Dental v3.6; Blender Foundation, B4D iBar™ module) software program are beneficial in many cases to develop an inner bar using the specialized software module to strengthen and support the suprastructure. Also, whenever the veneering materials need to be changed, this design facilitates the workflow [1, 2, 36, 46]. 

The PEEK bar recommended dimensions from previous literature were a minimum occlusal-cervical height of 5 mm, a minimum anterior buccolingual width of 4 mm [35–37]. The patient’s inter-arch space and clinical situation allowed for a bar framework design with an occlusal-cervical height of 5.1–6.6 mm and a buccolingual width of 5–6 mm. The minimum suprastructure-designed wall thickness was 2.5 mm according to the manufacturer [41], to ensure a strong design. Buccal and lingual finish lines were created with a width of 1 mm to facilitate measurements and margin drawing later in the study [34, 35]. Regarding the titanium bar, smaller dimensions can be used. However, the exact dimensions were adopted to standardize the size for both bars.

In this study, the average marginal gap between the suprastructure and titanium framework was (70.99 ± 6.53)µm, while the average marginal gap for PEEK was (101.87 ± 21.51)µm. Suprastructures created from scanning the titanium bar were more adapted than those created from scanning the PEEK bar. This may be because titanium scans are more true than PEEK scans, which is supported by studies reporting that scanning titanium renders more accurate STL files [25, 26]. 

Revilla-León et al. proposed that the dark color of the titanium scan body may be the underlying factor [47]. These results are verified by Baranowski et al. [48] Azevedo et al. declared that titanium scanbodies were significantly more accurate than PEEK scanbodies [49]. 

However, the values of both groups are within the clinically acceptable marginal gap range reported by McLean and Fraunhofer [50], which is ≤ 100–120 μm for CAD/CAM restorations [51]. 

The results were consistent with a study conducted by Bandiaky et al. [16] who reported that the mean ± standard deviation of the marginal fit was (95.03 ± 12.74)µm in the test group and (106.02 ± 14.51)µm in the control group. [16] Also, Kohorst et al. [52] reported a mean horizontal marginal discrepancy of 116.3 μm while testing the marginal accuracy of four-unit zirconia fixed dental prostheses. Another study conducted by Batson et al. [27] reported a horizontal marginal discrepancy of 113.8 ± 43.2 μm and 92.4 ± 20.6 μm for lithium disilicate and ceramic crowns.

Nano-ceramic-filled resins are 3D-printed definitive resins that can be utilized for full arch suprastructure fabrication and were chosen for this study for their modified physical and mechanical properties. They are commonly used for fabricating veneers, crowns, inlays, onlays, monolithic full arch restorations, partial dentures, and temporary bridges. This advanced biocompatible material delivers premium durability, patient comfort, and satisfying aesthetics for printing monolithic full dentures [28, 41]. 

This study did not primarily focus on comparing intraoral and lab scan STL files; nonetheless, as part of our quality control protocol, we assessed the trueness of intraoral scans relative to their matching desktop-scanned reference files. RMS and colour map analyses were performed to verify that discrepancies between intraoral and desktop scans of each bar remained within clinically acceptable limits.

Suprastructures were created from scanned substructures to enhance and perfect the fit between them [10, 13, 39]. Intraoral scanning was used to scan the bars in this study. If the bar was modified chairside, intraoral scanning captures this updated version. This approach reduces clinical steps and improves patient comfort. It also follows Medit’s recommended protocol, which includes scanning the soft tissue first and then the bar in situ to optimize the gingival contours of the suprastructures [53]. 

This study did not modify the titanium or PEEK suprastructures post-fabrication or before measurements. This was a deliberate choice to distinguish the accuracy of the digital operation and eradicate operator-induced variability. We could objectively assess the generated marginal fit and RMS values by avoiding manual modifications. After measurements, the titanium bar and its corresponding suprastructure were selected for the final restoration. The titanium group exhibited enhanced marginal adaptation and overall trueness. The suprastructures created from scanning the titanium bar were more 3-dimensionally accurate than those from PEEK. This may be because titanium bar scans were more accurate, leading to more accurate 3D-printed suprastructures [25]. which is reflected clinically with fewer occlusal inaccuracies that will lead to less chairside modifications.

The distribution of deviations was evaluated and visualized using colour maps [54–57]. While RMS offers a standardized assessment of overall deviation, it does not directly correlate with clinical outcomes. Localized discrepancies can have varying clinical implications: minor deviations on the intaglio surface are often compensable by luting cement. In contrast, over-contoured occlusal contacts and intaglio misfits may jeopardize restoration performance and result in failure.

Moreover, unless the color maps are observed, RMS measures the magnitude of deviation, failing to differentiate between positive and negative errors statistically [58]., with blue regions representing negative deviations (under-contoured areas) and red regions denoting positive deviations (over-contoured areas) [59]. Consequently, the interpretation must be supplemented by an assessment of color maps and an analysis of the distribution [60]. 

The 3D overall trueness is a valid tool for assessing 3D printed suprastructures. In a study conducted by Reich et al. [19], the trueness of 3D-printed occlusal devices was reported with a range of (122 ± 12)µm, which is similar to this study’s RMS findings of suprastructures’ trueness created from scanned titanium bars. However, the RMS of suprastructures created from PEEK bars was higher than (212.60 ± 54.76) µm, which deviated significantly from the reference scan. This huge deviation may be attributed to marginal gap results, as it was directly related to it; the titanium group had better marginal adaptation and better overall trueness results, too. In this study, all the variables were standardized: the printed resin, the 3 d printer, the anatomic form of the suprastructures, and the software program.

The variation in the 3 d trueness of the suprastructures could be directly related to the only available variable, the marginal gap. Batson et al. [27] linked the occlusion as a prosthesis quality determinant to the marginal gap, at which the accuracy of the suprastructure affected the occlusion, which was reflected by the amount of necessary occlusal adjustments.

This study is a single-arm clinical trial, with some limitations. Using a single clinical case design may inherently limit the generalizability of the results. A larger sample size would improve the findings’ statistical power and external validity. Nonetheless, this study design was deliberately selected to minimize inter-patient variability and to focus solely on the influence of the framework material, enabling a controlled comparison of the two framework materials under identical clinical conditions. Intraoral variables such as mucosal color, soft tissue reflectivity, salivary presence, temperature, humidity, and individual anatomical differences can influence scan accuracy and prosthesis fit. Limiting the study to a single patient is reported in previous literature [61, 62]. 

Our study focused on the marginal gap and 3D trueness of the scanned suprastructures, rather than the inherent strength of the restorative material. Further research on the mechanical properties of the material may be beneficial.

Reduced marginal gaps and enhanced 3D trueness of suprastructures produced from scanning titanium bars minimize adjustment time, leading to more accurate occlusion, increased passive fit, and, in comparison to PEEK substructures, may augment the long-term effectiveness and more predictable outcomes of full-arch implant prostheses.

## Conclusions

According to the findings of this research, the following conclusions were drawn:

1- Suprastructures created from scanning the titanium framework have better marginal adaptation and 3D trueness than those created from scanning the PEEK framework.

2- The marginal fit achieved for the 2 groups was within the range of clinical acceptance.

## Supplementary Information

Supplementary Material 1.

## Data Availability

The datasets generated and/or analysed during the current study are available in the following link. (https:/figshare.com/articles/dataset/Marginal_adaptation_and_three-dimensional_accuracy_of_two_different_substructure_framework_materials_for_full_arch_implant-supported_restorations/29589671).

## Abbreviations

3D: Three-dimensional
CAD-CAM: Computer-Aided Design - Computer-Aided Manufacture
IOS: IOS Intraoral Scanner
RMS: Root mean square
ISO: International Organisation for Standardisation
PFM: Porcelain fused to metal

## References

[1] Montero J, Macedo de Paula C, Albaladejo A. The Toronto prosthesis, an appealing method for restoring patients candidates for hybrid overdentures: a case report. J Clin Exp Dent. 2012;4(5):e309–312. 10.4317/jced.50877 PMC3892208 24455041

[2] Scarano A, Stoppaccioli M, Casolino T. Zirconia crowns cemented on titanium bars using CAD/CAM: a five-year follow-up prospective clinical study of 9 patients. BMC Oral Health. 2019;19(1):286. 31856799 10.1186/s12903-019-0988-x PMC6921470

[3] Cevik P, Schimmel M, Yilmaz B. New generation CAD-CAM materials for implant-supported definitive frameworks fabricated by using subtractive technologies. BioMed Res Int. 2022;2022:3074182. 35281596 10.1155/2022/3074182 PMC8906986

[4] Katsoulis J, Mericske-Stern R, Rotkina L, Zbären C, Enkling N, Blatz MB. Precision of fit of implant-supported screw-retained 10-unit computer-aided-designed and computer-aided-manufactured frameworks made from zirconium dioxide and titanium: an *in vitro* study. Clin Oral Implants Res. 2014;25(2):165–74. 23025489 10.1111/clr.12039

[5] Al-Meraikhi H, Yilmaz B, McGlumphy E, Brantley W, Johnston WM. In vitro fit of CAD-CAM complete arch screw-retained titanium and zirconia implant prostheses fabricated on 4 implants. J Prosthet Dent. 2018;119(3):409–16. 28720339 10.1016/j.prosdent.2017.04.023

[6] Dutton E, Ludlow M, Mennito A, Kelly A, Evans Z, Culp A, et al. The effect different substrates have on the trueness and precision of eight different intraoral scanners. J Esthet Restor Dent. 2020;32(2):204–18. 31568660 10.1111/jerd.12528

[7] Yilmaz B, Guo X, Schimmel M, Abou-Ayash S. Effect of industrial scanner and framework material interaction on the marginal gaps of CAD-CAM complete arch implant frameworks. J Prosthet Dent. 2023;130(5):723–30. 34998580 10.1016/j.prosdent.2021.10.013

[8] Dede D, Çakmak G, Donmez MB, Küçükekenci AS, Lu WE, Ni AA, et al. Effect of analysis software program on measured deviations in complete arch, implant-supported framework scans. J Prosthet Dent. 2024;132(1):211–8. 37596157 10.1016/j.prosdent.2023.06.028

[9] Nassar HI, Abdelaziz MS. Retention of bar clip attachment for mandibular implant overdenture. BMC Oral Health. 2022;22(1):227. 35681163 10.1186/s12903-022-02262-7 PMC9178882

[10] Lin WS, Chou JC, Metz MJ, Harris BT, Morton D. Use of intraoral digital scanning for a CAD/CAM-fabricated milled bar and superstructure framework for an implant-supported, removable complete dental prosthesis. J Prosthet Dent. 2015;113(6):509–15. 25862270 10.1016/j.prosdent.2015.01.014

[11] Papaspyridakos P, Rajput N, Kudara Y, Weber HP. Digital workflow for fixed implant rehabilitation of an extremely atrophic edentulous mandible in three appointments. J Esthet Restor Dent. 2017;29(3):178–88. 28316122 10.1111/jerd.12290

[12] Uribarri A, Bilbao-Uriarte E, Segurola A, Ugarte D, Verdugo F. Marginal and internal fit of CAD/CAM frameworks in multiple implant-supported restorations: scanning and milling error analysis. Clin Implant Dent Relat Res. 2019;21(5):1062–72. 31454146 10.1111/cid.12839

[13] Maló P, de Sousa ST, De Araújo Nobre M, Moura Guedes C, Almeida R, Roma Torres A, et al. Individual lithium disilicate crowns in a full-arch, implant-supported rehabilitation: a clinical report. J Prosthodont. 2014;23(6):495–500. 24495129 10.1111/jopr.12137

[14] Khaledi AAR, Vojdani M, Farzin M, Pirouzi S, Orandi S. The effect of sintering time on the marginal fit of zirconia copings. J Prosthodont. 2019;28(1):e285-9. 29314433 10.1111/jopr.12731

[15] Yucel MT, Aykent F, Avunduk MC. In vitro evaluation of the marginal fit of different all-ceramic crowns. J Dent Sci. 2013;8(3):225–30.

[16] Bandiaky ON, Clouet R, Le Bars P, Soueidan A, Le Guehennec L. Marginal and internal fit of five-unit zirconia-based fixed dental prostheses fabricated with digital scans and conventional impressions: a comparative in vitro study. J Prosthodont. 2023;32(9):846–53. 36627825 10.1111/jopr.13639

[17] Singla M, Padmaja K, Arora J, Shah A. Provisional restorations in fixed prosthodontics. Int J Dent Med Res. 2014;1(4):148–51.

[18] Ashour AM, El-Kateb MM, Azer AS. The effect of two preparation designs on the fracture resistance and marginal adaptation of two types of ceramic crowns using CAD/CAM technology (in vitro study). BMC Oral Health. 2024;24(1):1065. 39261857 10.1186/s12903-024-04742-4 PMC11391740

[19] Reich S, Berndt S, Kühne C, Herstell H. Accuracy of 3D-printed occlusal devices of different volumes using a digital light processing printer. Appl Sci. 2022;12(3):1576.

[20] Alghauli MA, Aljohani R, Almuzaini S, Aljohani W, Almutairi S, Alqutaibi AY. Accuracy, marginal, and internal fit of additively manufactured provisional restorations and prostheses printed at different orientations. J Esthet Restor Dent. 2025;37(4):934–49. 39503606 10.1111/jerd.13346

[21] Alghauli M, Alqutaibi AY, Wille S, Kern M. 3D-printed versus conventionally milled zirconia for dental clinical applications: trueness, precision, accuracy, biological and esthetic aspects. J Dent. 2024;144:104925. 38471580 10.1016/j.jdent.2024.104925

[22] Accuracy IS. of measurement methods and results—part 1: General principles and definitions. Geneva: International Organization for Standardization; 1994.

[23] Arnold C, Hey J, Schweyen R, Setz JM. Accuracy of CAD-CAM-fabricated removable partial dentures. J Prosthet Dent. 2018;119(4):586–92. 28709674 10.1016/j.prosdent.2017.04.017

[24] Cakmak G, Marques VR, Donmez MB, Lu WE, Abou-Ayash S, Yilmaz B. Comparison of measured deviations in digital implant scans depending on software and operator. J Dent. 2022;122:104154. 35526751 10.1016/j.jdent.2022.104154

[25] Emam NS, Khamis MM, Abdelhamid AM, Ezzelarab S. Digitization accuracy and scannability of different prosthodontic materials: an *in vitro* trial. J Prosthet Dent. 2023;130(2):e252251-8. 10.1016/j.prosdent.2023.05.032 37468368

[26] Gehrke P, Rashidpour M, Sader R, Weigl P. A systematic review of factors impacting intraoral scanning accuracy in implant dentistry with emphasis on scan bodies. Int J Implant Dent. 2024;10(1):20. 38691258 10.1186/s40729-024-00543-0 PMC11063012

[27] Batson ER, Cooper LF, Duqum I, Mendonça G. Clinical outcomes of three different crown systems with CAD/CAM technology. J Prosthet Dent. 2014;112(4):770–7. 24980739 10.1016/j.prosdent.2014.05.002

[28] Shin H, Kang YJ, Kim H, Kim JH. Effect of cement space settings on the marginal and internal fit of 3D printed definitive resin crowns. J Prosthet Dent. 2025;133(3):821–6. 37202234 10.1016/j.prosdent.2023.03.021

[29] Martinez-Rus F, Ferreiroa A, Ozcan M, Pradies G. Marginal discrepancy of monolithic and veneered all-ceramic crowns on titanium and zirconia implant abutments before and after adhesive cementation: a scanning electron microscopy analysis. Int J Oral Maxillofac Implants. 2013;28(2):480–7. 23527350 10.11607/jomi.2759

[30] Abduo J, Lyons K, Swain M. Fit of zirconia fixed partial denture: a systematic review. J Oral Rehabil. 2010;37(11):866–76. 20557435 10.1111/j.1365-2842.2010.02113.x

[31] International Organization for Standardization ISO 10477. Dentistry d polymer-based crown and bridge materials. 2022. Available at: https://www.iso.org/standard/80007.html.

[32] Grzebieluch W, Kowalewski P, Grygier D, Rutkowska-Gorczyca M, Kozakiewicz M, Jurczyszyn K. Printable and machinable dental restorative composites for CAD/CAM application-comparison of mechanical properties, fractographic, texture and fractal dimension analysis. Materials. 2021. 10.3390/ma14174919. 34501009 10.3390/ma14174919 PMC8434230

[33] Daher R, Ardu S, di Bella E, Krejci I, Duc O. Efficiency of 3D printed composite resin restorations compared with subtractive materials: evaluation of fatigue behavior, cost, and time of production. J Prosthet Dent. 2024;131(5):943–50. 36333176 10.1016/j.prosdent.2022.08.001

[34] Jivraj S. Graftless solutions for the edentulous patient. Springer Nature; 2023.

[35] Maló P, de Araújo Nobre M, Moura Guedes C, Almeida R, Silva A, Sereno N, et al. Short-term report of an ongoing prospective cohort study evaluating the outcome of full-arch implant-supported fixed hybrid polyetheretherketone-acrylic resin prostheses and the all-on-four concept. Clin Implant Dent Relat Res. 2018;20(5):692–702. 30110132 10.1111/cid.12662

[36] Mijiritsky E, Elad A, Krausz R, Ivanova V, Zlatev S. Clinical performance of full-arch implant-supported fixed restorations made of monolithic zirconia luted to a titanium bar: a retrospective study with a mean follow-up of 16 months. J Dent. 2023;137:104675. 37607658 10.1016/j.jdent.2023.104675

[37] de Araújo Nobre M, Moura Guedes C, Almeida R, Silva A, Sereno N. Hybrid polyetheretherketone (PEEK)-acrylic resin prostheses and the all-on-4 concept: a full-arch implant-supported fixed solution with 3 years of follow-up. J Clin Med. 2020. 10.3390/jcm9072187. 32664393 10.3390/jcm9072187 PMC7408851

[38] Finger C, Stiesch M, Eisenburger M, Breidenstein B, Busemann S, Greuling A. Effect of sandblasting on the surface roughness and residual stress of 3Y-TZP (zirconia). SN Appl Sci. 2020;2(10):1700.

[39] Rutkunas V, Gedrimiene A, Akulauskas M, Fehmer V, Sailer I, Jegelevicius D. In vitro and in vivo accuracy of full-arch digital implant impressions. Clin Oral Implants Res. 2021;32(12):1444–54. 34543478 10.1111/clr.13844

[40] El Gohary MK, Metwally MF, Shokry TE. Evaluation of vertical marginal gap of long span implant supported fixed dental prostheses fabricated with different CAD/CAM materials. Int J Med Sci Dent Res. 2022;5:21–36.

[41] EnvisionTEC GmbH. Instructions for Use – Flexcera® Smile Ultra+ (light-curable resin) [Internet]. Gladbeck: EnvisionTEC GmbH; 2025. Available from: https://dental.proto3000.com/wp-content/uploads/2022/04/Instruction-for-Use-Flexcera-Smile-Ultra-plus-rev.01.pdf. Accessed 06 Nov 2025.

[42] Kordi AWM, Salman AI, Metwally NA, Khamis MM. Evaluation of the masking ability, marginal adaptation, and fracture resistance of screw-retained lithium disilicate implant-supported crowns cemented to titanium bases versus preparable abutments. BMC Oral Health. 2023;23(1):613. 37649061 10.1186/s12903-023-03281-8 PMC10470172

[43] Rueden CT, Schindelin J, Hiner MC, DeZonia BE, Walter AE, Arena ET, et al. ImageJ2: imagej for the next generation of scientific image data. BMC Bioinformatics. 2017;18(1):529. 29187165 10.1186/s12859-017-1934-z PMC5708080

[44] Sidhom M, Zaghloul H, Mosleh IE, Eldwakhly E. Effect of different CAD/CAM milling and 3D printing digital fabrication techniques on the accuracy of PMMA working models and vertical marginal fit of PMMA provisional dental prosthesis: an in vitro study. Polymers. 2022;14(7):1285. 35406159 10.3390/polym14071285 PMC9003362

[45] Yilmaz B, Marques VR, Donmez MB, Cuellar AR, Lu WE, Abou-Ayash S, et al. Influence of 3D analysis software on measured deviations of CAD-CAM resin crowns from virtual design file: an in-vitro study. J Dent. 2022;118:103933. 34929340 10.1016/j.jdent.2021.103933

[46] Hobrink J, Zarb GA, Bolender CL, Eckert S, Jacob R, Fenton A, Mericske-Stern R. Prosthodontic treatment for edentulous patients: complete dentures and implant-supported prostheses. Elsevier Health Sciences; 2003.

[47] Revilla-León M, Young K, Sicilia E, Cho SH, Kois JC. Influence of definitive and interim restorative materials and surface finishing on the scanning accuracy of an intraoral scanner. J Dent. 2022;120:104114. 35358659 10.1016/j.jdent.2022.104114

[48] Baranowski JH, Stenport VF, Braian M, Wennerberg A. Effects of scan body material, length and top design on digital implant impression accuracy and usability: an *in vitro* study. J Adv Prosthodont. 2025;17(3):125–36. 40687194 10.4047/jap.2025.17.3.125 PMC12270716

[49] Azevedo L, Marques T, Karasan D, Fehmer V, Sailer I, Correia A, et al. Influence of implant scanbody material and intraoral scanners on the accuracy of complete-arch digital implant impressions. Int J Prosthodont. 2024;37(5):575–82. 37729480 10.11607/ijp.8565

[50] Flügge T, van der Meer WJ, Gonzalez BG, Vach K, Wismeijer D, Wang P. The accuracy of different dental impression techniques for implant-supported dental prostheses: a systematic review and meta-analysis. Clin Oral Implants Res. 2018;29(Suppl 16):374–92. 30328182 10.1111/clr.13273

[51] Akbar JH, Petrie CS, Walker MP, Williams K, Eick JD. Marginal adaptation of cerec 3 CAD/CAM composite crowns using two different finish line preparation designs. J Prosthodont. 2006;15(3):155–63. 16681497 10.1111/j.1532-849X.2006.00095.x

[52] Kohorst P, Brinkmann H, Li J, Borchers L, Stiesch M. Marginal accuracy of four-unit zirconia fixed dental prostheses fabricated using different computer-aided design/computer-aided manufacturing systems. Eur J Oral Sci. 2009;117(3):319–25. 19583762 10.1111/j.1600-0722.2009.00622.x

[53] https://support.medit.com/hc/en-us/articles/360020924592--colLab-The-substructure-of-an-implant-bar. MhccTsoaiboAa.

[54] Maeng J, Lim YJ, Kim B, Kim MJ, Kwon HB. A new approach to accuracy evaluation of single-tooth abutment using two-dimensional analysis in two intraoral scanners. Int J Environ Res Public Health. 2019. 10.3390/ijerph16061021. 30897832 10.3390/ijerph16061021 PMC6466129

[55] Lee JH, Yun JH, Han JS, Yeo IS, Yoon HI. Repeatability of intraoral scanners for complete arch scan of partially edentulous dentitions: an in vitro study. J Clin Med. 2019;8(8):1187. 31398851 10.3390/jcm8081187 PMC6722554

[56] Yang X, Lv P, Liu Y, Si W, Feng H. Accuracy of digital impressions and fitness of single crowns based on digital impressions. Materials. 2015;8(7):3945–57. 28793417 10.3390/ma8073945 PMC5455646

[57] Chen Y, Zhai Z, Li H, Yamada S, Matsuoka T, Ono S, et al. Influence of liquid on the tooth surface on the accuracy of intraoral scanners: an in vitro study. J Prosthodont. 2022;31(1):59–64. 33829613 10.1111/jopr.13358

[58] Chebib N, Imamura Y, El Osta N, Srinivasan M, Müller F, Maniewicz S. Fit and retention of complete denture bases: part II - conventional impressions versus digital scans: a clinical controlled crossover study. J Prosthet Dent. 2024;131(4):618–25. 36055812 10.1016/j.prosdent.2022.07.004

[59] Jang Y, Sim JY, Park JK, Kim WC, Kim HY, Kim JH. Evaluation of the marginal and internal fit of a single crown fabricated based on a three-dimensional printed model. J Adv Prosthodont. 2018;10(5):367–73. 30370028 10.4047/jap.2018.10.5.367 PMC6202428

[60] Revilla-León M, Meyer MJ, Özcan M. Metal additive manufacturing technologies: literature review of current status and prosthodontic applications. Int J Comput Dent. 2019;22(1):55–67. 30848255

[61] Hawksworth O, Chatters R, Julious S, Cook A, Biggs K, Solaiman K, et al. A methodological review of randomised n-of-1 trials. Trials. 2024;25(1):263. 38622638 10.1186/s13063-024-08100-1 PMC11020886

[62] Abdelhakim NA, Salazar‐Gamarra R, Segaan LG, Soliman IS. Evaluation of different technologies used for extraoral surface data acquisition for 3D facial scanning. Journal of Prosthodontics. 2025. 10.1111/jopr.14042 39930457

